# Does Hand-Predominance Have a Predominant Influence on Craniofacial Asymmetric and Anthropometric Analysis in Preadolescences?

**DOI:** 10.3390/diagnostics14212359

**Published:** 2024-10-23

**Authors:** Gloria Chen, Junior Chun-Yu Tu, Shih-Heng Chen, Emma Yuh-Jia Hsieh, Betty C. J. Pai, Ching-Yen Tsai, Pang-Yun Chou

**Affiliations:** 1 Department of Plastic and Reconstructive Surgery, Craniofacial Research Center, Chang Gung Memorial Hospital, Chang Gung University, Taoyuan 333, Taiwanjr_tu@hotmail.com (J.C.-Y.T.); shihheng@icloud.com (S.-H.C.); kingjack05@gmail.com (C.-Y.T.); 2 Division of Craniofacial Orthodontics, Department of Dentistry, Craniofacial Research Center, Chang Gung Memorial Hospital, Taoyuan 333, Taiwan; emma.jia@gmail.com (E.Y.-J.H.); pai0072@cgmh.org.tw (B.C.J.P.); 3 Department of Mechanical Engineering, Chang Gung University, Taoyuan 333, Taiwan

**Keywords:** 3D stereophotogrammetry, craniofacial asymmetry, laterality, school-aged children, hand-predominance

## Abstract

Background: Although the human body generally exhibits bilateral symmetry, achieving perfect symmetry is exceedingly uncommon. During preadolescent development, a face that approximates symmetry is considered both aesthetically and functionally ideal. This study aimed to investigate the relationship between craniofacial traits and hand predominance, using three-dimensional (3D) stereophotogrammetry to discern whether facial soft-tissue characteristics are correlated with hand preference. Materials and Methods: The study involved children aged 9 and 10 years who were free from any diagnosed craniofacial anomalies. Three-dimensional stereophotogrammetry was conducted to analyze their facial morphology, and 37 distinct anatomical landmarks were manually identified using a MATLAB-developed program. Results: A total of 188 Taiwanese children participated in the study. All participants were healthy, with a mean age of 9.79 years. Among them, 93.1% (175) were right-hand predominant, and 6.9% (13) were left-hand predominant. There were no significant differences in linear parameters or facial asymmetry between right-hand-predominant and left-hand-predominant participants (*p* > 0.05). However, a consistent trend toward right laterality, especially in the right lateral frontal region of the cranium, was observed, as illustrated by heat maps of the average three-dimensional model. Conclusions: The study found no association between facial morphology and hand predominance. A normal asymmetry with a rightward tendency was noted in children aged 9 to 10 years, which was particularly notable in the lateral frontal region of the head.

## 1. Introduction

Facial symmetry is widely recognized as a fundamental element of facial harmony. However, asymmetry is prevalent in the majority of bilateral vertebrates, including humans [1]. Although the human body typically develops with bilateral symmetry, achieving perfect symmetry is rare [1,2]. Most individuals exhibit minor craniofacial asymmetries, which can be attributed to both genetic factors and external influences [1]. Only pronounced disproportions and significant facial asymmetries are typically noticed, making facial asymmetry more of an expectation than an exception [1,2,3].

During preadolescent development, a nearly symmetric face is considered both aesthetically and functionally ideal [1,4]. Surgical interventions are often required for individuals with congenital craniofacial anomalies or notable bilateral discrepancies [2]. Understanding the trends in craniofacial morphology and symmetry among healthy children is imperative for early interventions [2]. Although a right-sided facial laterality has often been noted in the literature, some dissenting opinions support left-sided facial predominance [3,4,5,6]. Nonetheless, reports indicate a consistent pattern in facial laterality rather than random occurrences.

Hand predominance refers to a preference for using one hand over the other, as well as hand movement requiring precise coordination, force calibration, and timely execution [7]. Studies have revealed distinct differences between right- and left-hand predominance traits. For example, Nicholls et al.’s finding on hand-predominance-related differences in general cognitive ability and Grimshaw et al.’s work on identifying correlation between hand predominance and personality traits [8,9]. However, the link between hand predominance and facial morphology remains unclear, with most conclusions drawn from visual observations [10,11].

Although numerous studies have investigated facial asymmetry, the majority have relied on two-dimensional images and photographs [3,4,5,6,12,13,14]. To date, no research has examined the association between hand predominance and craniofacial morphology using three-dimensional (3D) soft-tissue features. Employing three-dimensional soft-tissue imaging overcomes the limitations inherent in two-dimensional techniques, especially those related to capturing the intricate three-dimensional contours and depths of facial features [15,16,17]. Therefore, this study aims to explore the relationship between hand predominance craniofacial traits using three-dimensional stereophotogrammetry. Specifically, we sought to answer the following research question: Is there an association between hand predominance and facial soft-tissue morphology in healthy children?

## 2. Materials and Methods

This study (identification number: 2201900438B0) received approval from the Ethics Committee for Human Research at Taoyuan Chang Gung Memorial Hospital, Taiwan.

This study enrolled healthy Taiwanese children aged 9 to 10 years. Inclusion criteria required participants to be free of diagnosed craniofacial abnormalities, previous surgeries, or any history of craniofacial trauma. Children who did not meet these criteria were excluded from the study. All participants underwent 3D stereophotogrammetry, and 3D images were captured using the 3dMD head system (3dMD LLC, Atlanta, GA, USA) [18]. The system is equipped with multiple modular machine vision cameras. During image acquisition, each participant was instructed to maintain a consistent position and adopt a neutral facial expression.

**Figure 1:**
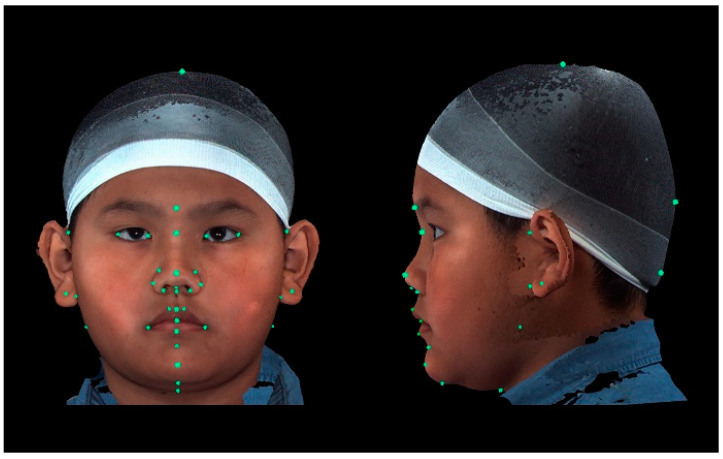
Placement of landmarks in frontal and left lateral profile views. A total of 37 distinct facial soft-tissue structures were placed manually as landmarks for the measurement-based analyses.

**Figure 2:**
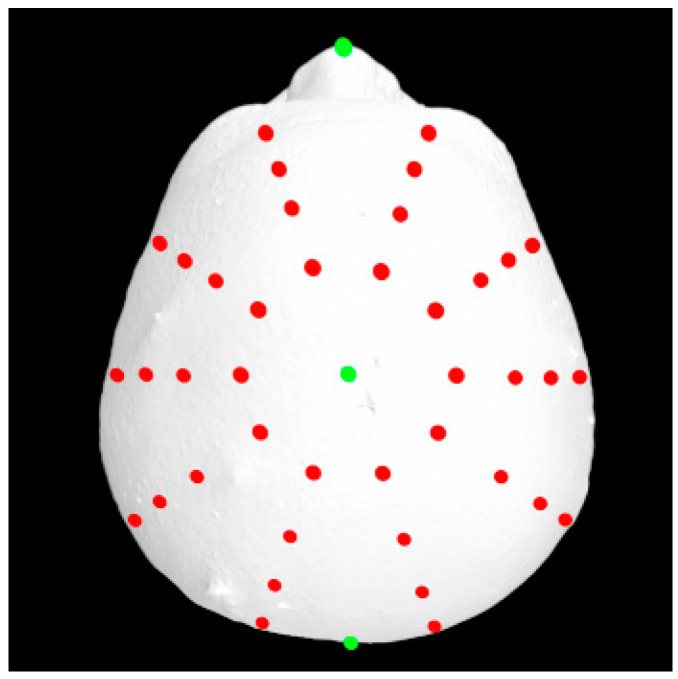
An additional 40 digitally distributed landmarks (red) for asymmetry analyses.

A total of 37 distinct landmarks on facial soft-tissue structures were manually identified in each 3D image using 3dMD software (v. 2.2; 3dMD, Atlanta, GA, USA; Figure 1 and Table 1). To examine craniofacial asymmetry, 4 parallel planes, each descending 10° from the previous plane, were established from the skull apex [18]. Ten landmarks were evenly distributed around the Y-axis in each plane, and 40 additional landmarks were digitally placed around the skull (Figure 2). The 3D Cartesian coordinate system’s origin was established by projecting the pronasale onto the line connecting the bilateral tragus [18].

Facial characteristics were analyzed using 14 parameters: 9 linear and 5 angular parameters.

**Figure 3:**
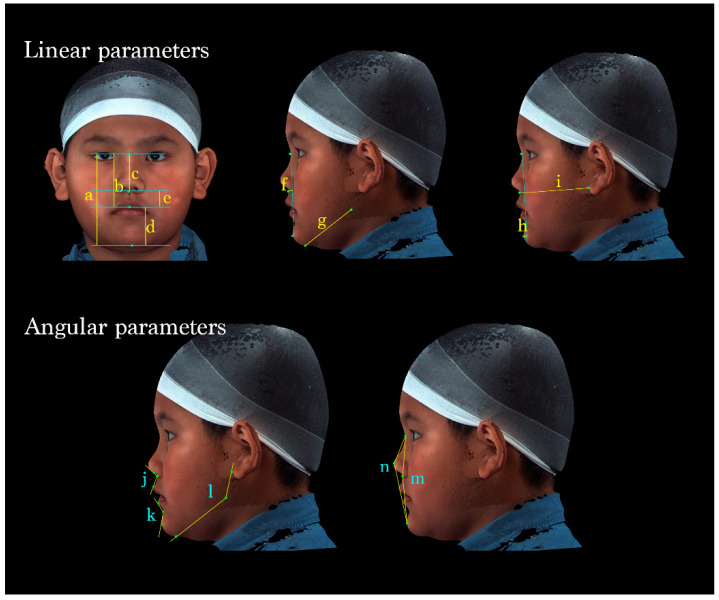
A total of 14 parameters were measured. (Upper left) Vertical dimensions of the face: (a) N-Gn, (b) N-Sto, (c) N-Sn, (d) Sto-Gn, and (e) Sn-Sto. (Middle) (f) Sn to N-Pg (position of the subnasale relative to the soft-tissue nasion-pogonion plane), (g) Go-Gn (mandibular length). (Upper right) (h) Pg to N-B (anteroposterior position of the pogonion), (i) Sn- OBi (middle facial depth). (Lower left) (j) ∠Cm-Sn-Ls (nasolabial angle); (k) ∠Li-B-Pg (labiomental angle); (l) ∠OBi-Go-Gn (gonial angle). (Lower right) (m) ∠N-Sn-Pg (Facial convexity); (n) ∠N-PRn-Pg (full soft-tissue convexity).

The vertical dimension of the face was evaluated by measuring the total facial, midface, total nasal, lower facial, and labiomental vertical lengths. The anteroposterior position of the subnasale, length of the mandibular body, sagittal position of the pogonion, and bilateral midface depth were measured and used in comprehensive linear analyses. Moreover, the nasolabial angle, labiomental angle, gonial angle, facial convexity, and full soft-tissue convexity were measured and used in angular assessments (Table 2 and Figure 3).

MATLAB (v. 9.11, MathWorks, Natick, MA, USA) was used to design a customized program for creating a perfectly symmetrical craniofacial template.

**Figure 4:**
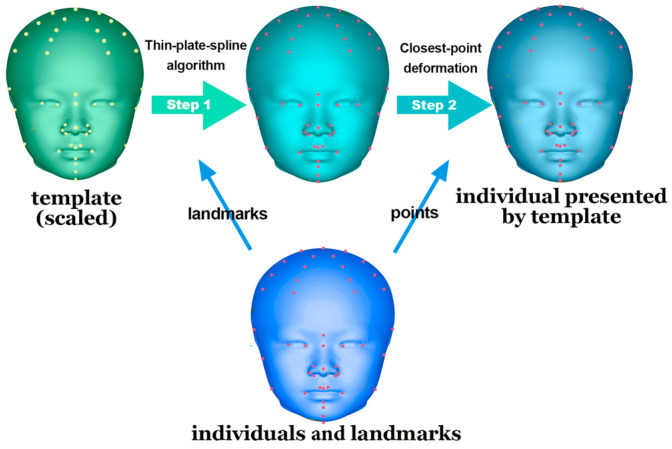
Application of the template for alignment. Thin-plate spline algorithm and closest-point deformation were conducted on a perfectly symmetrical template. The template was transformed based on the basis of corresponding landmarks, precisely presenting the subjects.

This template was aligned with each participant’s 3D soft-tissue scan after size adjustment. Subsequently, a thin-plate spline algorithm was employed for analysis based on the paired landmarks. Finally, a closest-point deformation algorithm was used to identify the corresponding points between the template and the 3D scan on the basis of Euclidean distance (Figure 4).

Standardized head measurements were obtained using the Farkas system of craniofacial anthropometry, with measurements being taken of the head transverse width, anteroposterior length, and head height. Laterality was determined by calculating the distances between each of the paired points and the origin on the 3D Cartesian coordinate system; it was determined by subtracting the distance between the left point and the origin from the distance between the right point and the origin. Participants with a predominantly positive difference were inferred to exhibit right head laterality.

Asymmetry was evaluated by comparing the distances of each paired point on the left and right sides from the origin.

**Figure 5:**
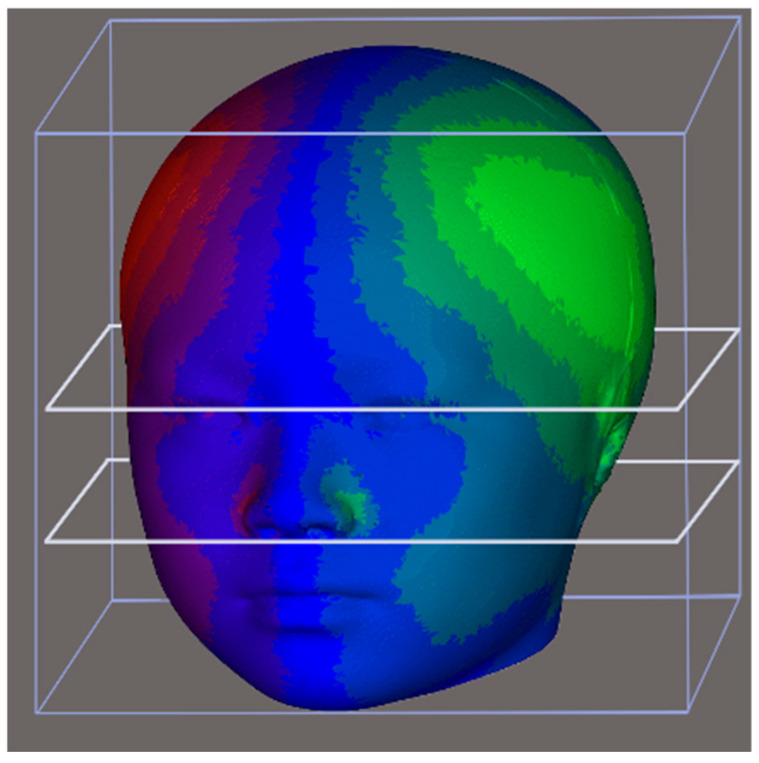
The 3D model was divided into upper, middle, and lower thirds. The upper third was defined as the area above the nasion, the middle third ranged from the axial plane through the nasion to the axial plane through the subnasale, and the lower third was below subnasale.

The original 3D model was segmented into upper, middle, and lower facial regions. The upper region was defined as the section above the nasion; the middle region extended from the axial plane, passed through the nasion, and continued to the subnasale; and the lower region encompassed the area below the subnasale (Figure 5).

All statistical analyses were conducted using SPSS (v27.0; IBM, Armonk, NY, USA). The associations between laterality, facial morphological traits, and asymmetry were analyzed using an unpaired 2-sample *t* test. A *p* value of <0.05 was considered statistically significant.

## 3. Results

Facial analyses were conducted on 188 3D images of healthy Taiwanese children who were recruited from local elementary schools; they were aged 9 to 10 years (mean age: 9.79 years). Among the participants, 94 were male and 94 were female children. In addition, 175 exhibited right-hand predominance, and 13 exhibited left-hand predominance (Table 3). All 3D images met the predefined criteria.

No significant differences in linear or angular facial parameters were observed between the children with right- and left-hand predominance (*p* > 0.05; Table 4).

Among the participants with right-hand predominance, the mean head transverse width, anteroposterior length, height, and volume were 226.12 mm, 181.10 mm, 162.51 mm, and 3497.22 mm^3^, respectively. Among the children with left-hand predominance, the mean head transverse width, anteroposterior length, height, and volume were 225.87 mm, 182.29 mm, 159.38 mm, and 3483.12 mm^3^, respectively. These results indicate that the two groups differed significantly only in the volume of the upper third of the head (*p* = 0.03).

The overall asymmetry values for the skull and face were 2.22 and 0.92 mm, respectively, among the participants with right-hand predominance, and were 2.41 and 0.95 mm, respectively, among those with left-hand predominance. Accordingly, the two groups did not differ significantly in terms of overall asymmetry (*p* > 0.05; Table 5).

**Figure 6:**
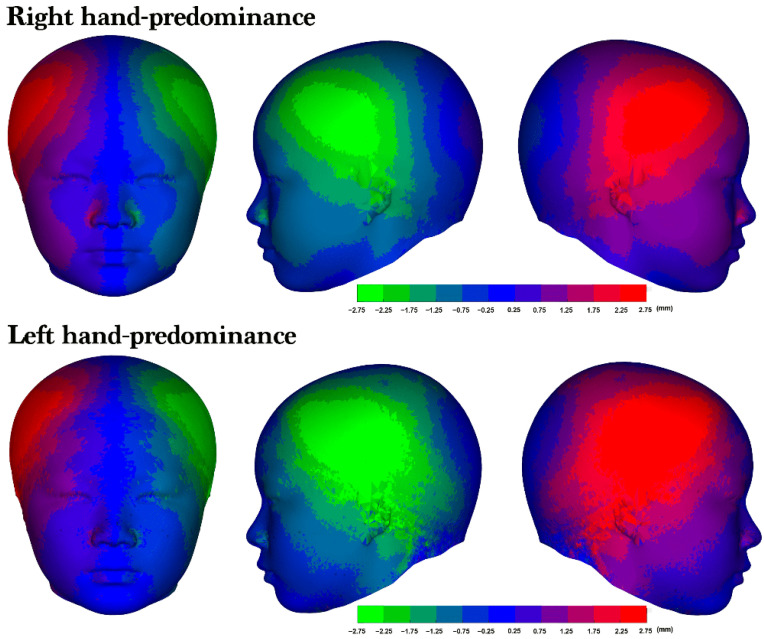
The average 3D model and laterality. An average asymmetry heat map of the right- and left-hand predominance groups. From left to right: frontal view, left lateral profile view, and right lateral profile view of the 3D heat map for each group. The colors represent the corresponding levels of asymmetry. The more vivid the color displayed in an area, the greater the degree of asymmetry observed.

Right laterality was predominant among both the participants with right-hand predominance (76.6%) and those with left-hand predominance (69.9%; Table 4). Furthermore, no significant differences in facial asymmetry were identified across the upper, middle, and lower 3D models; however, the heat maps of the average 3D models revealed a right-side protrusion in the cranium and face (Figure 5). Although both groups exhibited right laterality, the heat maps revealed a distinct asymmetry in the right lateral frontal region, with the participants with left-hand predominance having a more pronounced area of asymmetry (Figure 6).

## 4. Discussion

Previous attempts to understand facial laterality and asymmetry in humans have produced conflicting results [3,4,5,6]. In the present study, we conducted noninvasive 3D stereophotogrammetry on 188 children aged 9 to 10 years and observed a tendency toward right laterality. However, there were no significant differences in facial asymmetry between participants with right-hand predominance and those with left-hand predominance.

We chose to recruit children aged 9 to 10 years because they are likely to have stable craniofacial features, due in part to the full emergence of upper and lower permanent incisors at this age [19,20]. Furthermore, this age group has a lower likelihood of having acquired facial disfigurements. Previous studies using 2D facial photographs, cephalograms, and stereophotogrammetry have reported that neither sex nor age significantly influenced facial asymmetry [1,3,4,5,6,12,13,14]. By employing 3D stereophotogrammetry, we were able to minimize radiation exposure and achieved greater accuracy in pinpointing specific landmarks and constructing the 3D Cartesian coordinate system [15,16,17].

Hand predominance, the most extensively studied aspect of functional lateralization or brain asymmetry, plays a prominent role in performing daily activities. In general, hand predominance is considered to arise due to functional hemispheric asymmetry [21]. However, the neurobiological mechanism underlying hand predominance remains a topic of debate. Klöppel et al. used functional magnetic resonance imaging to observe movement-related activation of the primary sensorimotor cortex, supplementary motor area, and the caudal dorsal premotor cortex [22]. Nicholls et al. found a subtle but significant association between general cognitive ability and hand predominance, with individuals showing strong hand preference displaying slightly lower cognitive scores [8]. Furthermore, Grimshaw et al. found a psychological aspect to hand predominance, noting a curvilinear relationship wherein individuals with mixed hand predominance exhibited lower levels of extraversion [9]. Approximately 90% of people exhibit right-hand dominance and left-hemispheric dominance in performing manual tasks. However, the prevalence of left-hand dominance ranges from 0.5% and 24%, depending on the culture [23]. Notably, significant differences in the prevalence of left-hand predominance exist between White and East Asian populations [24].

During elementary school, socialization plays a pivotal role in a child’s socioemotional development. However, children with prominent craniofacial asymmetry might experience peer pressure due to societal perceptions [25]. Early identification of facial growth patterns can enable timely intervention by dental orthopedic and craniofacial specialists [2]. The literature has yet to achieve a consensus on the influence of facial laterality. Although some studies, such as those by Hsu et al. and Talisman et al., have reported on the prevalence of right facial laterality [4,5], the associations of hand predominance with facial morphology and laterality remain underexplored.

Though laterality often accompanies facial asymmetry, our study found no difference in facial asymmetry between the two groups. Notably, right laterality was observed regardless of hand preference. These findings align with previous research, but we also noted a prominence in the lateral frontal region in preadolescents. This contrasts with earlier studies reporting right-side dominance in the middle and lower faces of adults [3]. In light of these observations, we have initiated a longitudinal investigation at our institution to monitor changes in craniofacial morphology, especially considering potential changes during puberty [26].

Haraguchi et al. analyzed orthodontic patients using 2D facial photographs to measure hemiface and chin deviation. They revealed a consistent tendency toward right laterality across all ages and sexes. As participants experienced prepubertal and pubertal growth, the frequency of right laterality decreased [3]. However, a slight increase in left laterality was observed, which they hypothesized could be influenced by functional asymmetries, such as differential muscle mobility in facial expressions and habitual mastication [27].

Facial expressions produced on the left side of the face tend to be more pronounced, as most facial muscles receive innervation from the contralateral hemisphere [3,11,28,29]. Despite the apparent contradiction between muscle laterality and asymmetry, Okamoto et al. proposed that the laterality of facial expressions, especially smiles, is independent of hand predominance. This may be due to differences in neuronal pathways [11].

Myriad factors influence facial characteristics and asymmetry. These features often mirror the internal morphology of craniofacial structures, including muscles, fat pads, bones, and teeth alignment [4]. As a result, most studies conclude that environmental disturbances and hereditary factors primarily drive the development of facial characteristics [1,3,4,5,6,12,13,14]. Functional and developmental elements, such as habitual mastication, malocclusion, breathing patterns, nutrition, and oral habits, also shape an individual’s facial appearance [3,30]. By focusing our analysis on school-aged children, we minimized the influence of acquired facial characteristics that develop with age.

Hand predominance is a continuous variable, not a dichotomous feature [15]. Traditional categorizations of right- and left-hand predominance oversimplify this concept. The prevailing belief is that hand predominance stems from functional hemispheric asymmetry [7]. However, studies have shown distinct differences in brain activity patterns. For example, Siebner et al. observed a mirrored activation pattern in the brain hemisphere contralateral to the dominant hand in individuals without cross-dominance [7]. Similarly, Grabowska et al. reported that movements of the non-dominant hand elicited greater activation in the ipsilateral hemisphere [31]. These findings on facial asymmetry highlight functional and structural variations between the cerebral hemispheres. This suggests that the subtle inherent asymmetry seen in the face may arise from differences in the brain and skull base [32].

## 5. Limitations

This study has certain limitations that should be acknowledged. One limitation is the unequal sample size of participants with left-hand predominance compared to those with right-hand predominance. Although the overall prevalence of left-hand predominance was reported to be low in East Asian populations [23], the small number of left-hand predominant participants in this study may not be truly representative, which weakens the statistical power and limits the generalizability of our findings. Additionally, while 3D imaging is a reliable and precise technique frequently used in craniofacial studies, challenges can arise when areas imaging areas obscured by hair, making it difficult to accurately define the underlying soft-tissue surface in the posterior skull region.

## 6. Conclusions

Based on our 3D imaging study, hand predominance did not significantly influence facial asymmetry in healthy children. However, we observed a consistent right-sided facial laterality, with a marked protrusion in the lateral frontal region of the cranium.

## Figures and Tables

**Table 1:** Thirty-seven recognizable anatomical landmarks identified on 3D images.

Labels	Landmarks	Descriptions
Ala-l	Left alare	Most lateral point of the left alar
Ala-r	Right alare	Most lateral point of the right alar
As-l	Alar superior point, left	Left facial insertion of the alar
As-r	Alar superior point, right	Right facial insertion of the alar
B	Sublabiale	The most posterior midpoint of the labiomental soft-tissue contour
C	Columella	Midpoint of the columella crest
Cer	Cervical	Midpoint of the subhyoid depression
Ch-l	Cheilion, left	Labial commissure on the left
Ch-r	Cheilion, right	Labial commissure on the right
Cph-l	Left, crista philtra	The crossing of the vermilion line and elevated margin of the philtrum on the left
Cph-r	Right, crista philtra	The crossing of the vermilion line and elevated margin of the philtrum on the right
En-l	Endocanthion, left	Inner commissure of the left eye
En-r	Endocanthion, right	Inner commissure of the right eye
Ex-l	Exocanthion, left	Outer commissure of the left eye
Ex-r	Exocanthion, right	Outer commissure of the right eye
G	Glabella	Most anterior midpoint of the fronto-orbital soft-tissue contour
Gn	Gnathion	The most inferior midpoint of the chin
Go-l	Gonion, left	The most lateral point of the soft-tissue contour of the left mandibular angle
Go-r	Gonion, right	The most lateral point of the soft-tissue contour of the right mandibular angle
L-l	Left lobule	Center of the left earlobe
L-r	Right lobule	Center of the right earlobe
Li	Labiale inferius	Midpoint of the vermilion line of the lower lip
Ls	Labiale superius	Midpoint of the vermilion line of the upper lip
N	Nasion	Most posterior midpoint of the frontonasal soft-tissue contour
Nb-l	Nostril base point, left	Lowest point of the left nostril
Nb-r	Nostril base point, right	Lowest point of the right nostril
O	Occipital	The most anterior point at the occipital region of the head
OBi-l	Otobasian inferius, left	Left earlobe attachment point to the cheek
OBi-r	Otobasian inferius, right	Right earlobe attachment point to the cheek
OBs-l	Otobasion superius, left	Attachment point of the left helix in the temporal region
OBs-r	Otobasion superius, right	Attachment point of the right helix in the temporal region
Op	Opisthocranion	Most posterior point of the head
Pg	Pogonion	The most anterior midpoint of the chin
PRn	Pronasale	Most anterior midpoint of the nasal tip
Sn	Subnasale	Midpoint of the nasolabial soft-tissue contour between the columella crest and upper lip
Sto	Stomion	Midpoint of the horizontal labial fissure
V	Vertex	Highest point of the head

**Table 2:** List of 14 (9 linear and 5 angular) parameters included in the image analysis.

Measurements	Label	
Linear		
N-Gn	a	Total facial vertical dimension
N-Sto	b	Mid-facial vertical dimension
N-Sn	c	Total nasal vertical dimension
Sto-Gn	d	Lower facial vertical dimension
Sn-Sto	e	Upper lip vertical dimension
Sn to N-Pg	f	Position of the subnasale relative to the soft-tissue nasion-pogonion plane
Go-Gn	g	Mandibular length
Pg to N-B	h	Anteroposterior position of the pogonion
Sn-OBi	i	Middle facial depth
Angular		
∠Cm-Sn-Ls	j	Nasolabial angle
∠Li-B-Pg	k	Labiomental angle
∠OBi-Go-Gn	l	Mandibular angle
∠N-Sn-Pg	m	Facial convexity angle
∠N-PRn-Pg	n	Full soft-tissue convexity angle

**Table 3:** Demographic information of participants included in the study.

Demographic Characteristics	Value
Mean age	9.79
Gender	
Male	94 (50.0%)
Female	94 (50.0%)
Hand predominance	
Right	175 (93.1%)
Left	13 (6.9%)

**Table 4:** Mean linear and angular measurements with their standard deviations for different hand predominance in participants (*N* = 188; mean age = 9.79 years).

Measurements	Right-Hand Predominance (*n* = 175)	Left-Hand-Predominance (*n* = 13)	*p* Value
Linear			
N-Gn	102.62 ± 5.50	104.51 ± 8.46	0.44
N-Sn	42.58 ± 2.96	43.85 ± 2.38	0.10
Sto-Gn	43.33 ± 4.25	43.45 ± 6.25	0.08
Sn-Sto	20.34 ± 2.28	21.33 ± 2.51	0.18
N-Sto	62.63 ± 3.71	64.83 ± 4.5	0.68
Sn to N-Pg	6.27 ± 2.04	6.70 ± 1.92	0.94
Go-Gn	60.95 ± 4.22	59.45 ± 4.99	0.31
Pg to N-B	2.18 ± 2.00	1.87 ± 1.22	0.41
Sn-OBi	104.22 ± 5.02	104.66 ± 5.82	0.80
Angular			
∠Cm-Sn-Ls	105.88 ± 10.71	104.68 ± 10.17	0.4
∠Li-B-Pg	138.96 ± 15.27	136.16 ± 11.01	0.48
∠OBi-Go-Gn	146.09 ± 4.06	147.33 ± 3.79	0.44
∠N-Sn-Pg	163.23 ± 9.39	162.77 ± 5.09	0.77
∠N-PRn-Pg	137.05 ± 3.80	135.81 ± 4.03	0.30

**Table 5:** Mean craniofacial norms, head, facial asymmetry, and laterality with their standard deviations for different hand predominance in participants (*N* = 188; mean age = 9.79 years).

Parameters	Right-Hand Predominance (*n* = 175)	Left-Hand Predominance (*n* = 13)	*p* Value
Craniofacial norms			
Head height (mm)	226.12 ± 9.72	225.87 ± 15.29	0.95
Anteroposterior length (mm)	181.10 ± 8.75	182.29 ± 7.78	0.61
Head width (mm)	162.51 ± 11.46	159.38 ± 11.80	0.37
Asymmetry parameters			
Skull (mm)	2.22 ± 1.03	2.41 ± 1.79	0.71
Face (mm)	0.92 ± 0.45	0.95 ± 0.44	0.78
Right face laterality (%)	76.6	69.2	

## Data Availability

The data and materials can be shared upon the agreement of the corresponding author.

## References

[1] Shah S.M., Joshi M.R. (1978). An assessment of asymmetry in the normal craniofacial complex. Angle Orthod..

[2] Urban S.D., Waite P.D. (2005). Management of Facial Asymmetry. Am. J. Cosmet. Surg..

[3] Haraguchi S., Iguchi Y., Takada K. (2008). Asymmetry of the face in orthodontic patients. Angle Orthod..

[4] Talisman R., Arnon O., Weinberger A. (2022). Facial asymmetry, the right-side dominance: A retrospective analysis of 315 consecutive series of patients. JPRAS Open.

[5] Hsu C.K., Hallac R.R., Denadai R., Wang S.-W., Kane A.A., Lo L.-J., Chou P.-Y. (2019). Quantifying normal head form and craniofacial asymmetry of elementary school students in Taiwan. J. Plast. Reconstr. Aesthet. Surg..

[6] Ferrario V.F., Sforza C., Miani A., Serrao G. (1995). A three-dimensional evaluation of human facial asymmetry. J. Anat..

[7] Siebner H.R., Limmer C., Peinemann A., Drzezga A., Bloem B.R., Schwaiger M., Conrad B. (2002). Long-term consequences of switching handedness: A positron emission tomography study on handwriting in “converted” left-handers. J. Neurosci..

[8] Nicholls M.E., Chapman H.L., Loetscher T., Grimshaw G.M. (2010). The relationship between hand preference, hand performance, and general cognitive ability. J. Int. Neuropsychol. Soc..

[9] Grimshaw G.M., Wilson M.S. (2013). A sinister plot? Facts, beliefs, and stereotypes about the left-handed personality. Laterality.

[10] Borod J.C., Caron H.S., Koff E. (1981). Asymmetry of facial expression related to handedness, footedness, and eyedness: A quantitative study. Cortex.

[11] Okamoto H., Haraguchi S., Takada K. (2010). Laterality of asymmetry in movements of the corners of the mouth during voluntary smile. Angle Orthod..

[12] Farkas L.G., Posnick J.C., Hreczko T.M. (1992). Anthropometric growth study of the head. Cleft Palate Craniofacial J..

[13] Farkas L.G., Deutsch C.K. (1996). Anthropometric determination of craniofacial morphology. Am. J. Med. Genet..

[14] Ferrario V.F., Sforza C., Ciusa V., Dellavia C., Tartaglia G.M. (2001). The effect of sex and age on facial asymmetry in healthy subjects: A cross-sectional study from adolescence to mid-adulthood. J. Oral Maxillofac. Surg..

[15] Ghoddousi H., Edler R., Haers P., Wertheim D., Greenhill D. (2007). Comparison of three methods of facial measurement. Int. J. Oral Maxillofac. Surg..

[16] Nord F., Ferjencik R., Seifert B., Lanzer M., Gander T., Matthews F., Rücker M., Lübbers H.-T. (2015). The 3dMD photogrammetric photo system in cranio-maxillofacial surgery: Validation of interexaminer variations and perceptions. J. Craniomaxillofac. Surg..

[17] Cho M.J., Hallac R.R., Ramesh J., Seaward J.R., Hermann N.V., Darvann T.A., Lipira A., Kane A.A. (2018). Quantifying Normal Craniofacial Form and Baseline Craniofacial Asymmetry in the Pediatric Population. Plast. Reconstr. Surg..

[18] Chen G., Hsieh E.Y.-J., Chen S.-H., Pai B.C.J., Tsai C.-Y., Wang S.-W., Chou P.-Y. (2023). Occlusion-Based Three-Dimensional Craniofacial Anthropometric and Symmetric Evaluation in Preadolescences: A Comparative COHORT Study. J. Clin. Med..

[19] Subtelny J.D. (1959). A longitudinal study of soft tissue facial structures and their profile characteristics, defined in relation to underlying skeletal structures. Am. J. Orthod..

[20] Baume L.J. (1950). Physiological tooth migration and its significance for the development of occlusion. I. The biogenetic course of the deciduous dentition. J. Dent. Res..

[21] Ocklenburg S., Beste C., Güntürkün O. (2013). Handedness: A neurogenetic shift of perspective. Neurosci. Biobehav. Rev..

[22] Klöppel S., van Eimeren T., Glauche V., Vongerichten A., Münchau A., Frackowiak R.S., Büchel C., Weiller C., Siebner H.R. (2007). The effect of handedness on cortical motor activation during simple bilateral movements. Neuroimage.

[23] Geuze R.H., Schaafsma S.M., Lust J.M., Bouma A., Schiefenhövel W., Groothuis T.G. (2012). Plasticity of lateralization: Schooling predicts hand preference but not hand skill asymmetry in a non-industrial society. Neuropsychologia.

[24] Peters M., Reimers S., Manning J.T. (2006). Hand preference for writing and associations with selected demographic and behavioral variables in 255,100 subjects: The BBC internet study. Brain Cogn..

[25] Geckeler K.C., Barch D.M., Karcher N.R. (2022). Associations between social behaviors and experiences with neural correlates of implicit emotion regulation in middle childhood. Neuropsychopharmacology.

[26] Primozic J., Perinetti G., Contardo L., Ovsenik M. (2017). Facial soft tissue changes during the pre-pubertal and pubertal growth phase: A mixed longitudinal laser-scanning study. Eur. J. Orthod..

[27] Khamnei S., Sadat-Ebrahimi S.R., Salarilak S., Oskoee S.S., Houshyar Y., Shakouri S.K., Salekzamani Y., Zamanlu M. (2019). Manifestation of hemispheric laterality in chewing side preference and handedness. Bioimpacts.

[28] Dimberg U., Petterson M. (2000). Facial reactions to happy and angry facial expressions: Evidence for right hemisphere dominance. Psychophysiology.

[29] Indersmitten T., Gur R.C. (2003). Emotion processing in chimeric faces: Hemispheric asymmetries in expression and recognition of emotions. J. Neurosci..

[30] Launonen A.M., Vuollo V., Aarnivala H., Heikkinen T., Pirttiniemi P., Valkama A.M., Harila V. (2023). A longitudinal study of facial asymmetry in a normal birth cohort up to 6 years of age and the predisposing factors. Eur. J. Orthod..

[31] Grabowska A., Gut M., Binder M., Forsberg L., Rymarczyk K., Urbanik A. (2012). Switching handedness: fMRI study of hand motor control in right-handers, left-handers and converted left-handers. Acta Neurobiol. Exp..

[32] Pirttiniemi P. (1998). Normal and increased functional asymmetries in the craniofacial area. Acta Odontol. Scand..

